# Supplementary material for: Poly (ADP-Ribose) Polymerase Inhibitor Olaparib-Resistant BRCA1-Mutant Ovarian Cancer Cells Demonstrate Differential Sensitivity to PARP Inhibitor Rechallenge
Source: Cells. 2024 Nov 7;13(22):1847. doi: 10.3390/cells13221847 (PMC11592949; doi:10.3390/cells13221847)
Supplement: Supplementary file 1 [file cells-13-01847-s001.zip › cells-3229546-supplementary.pdf]

Supplementary figures and legends

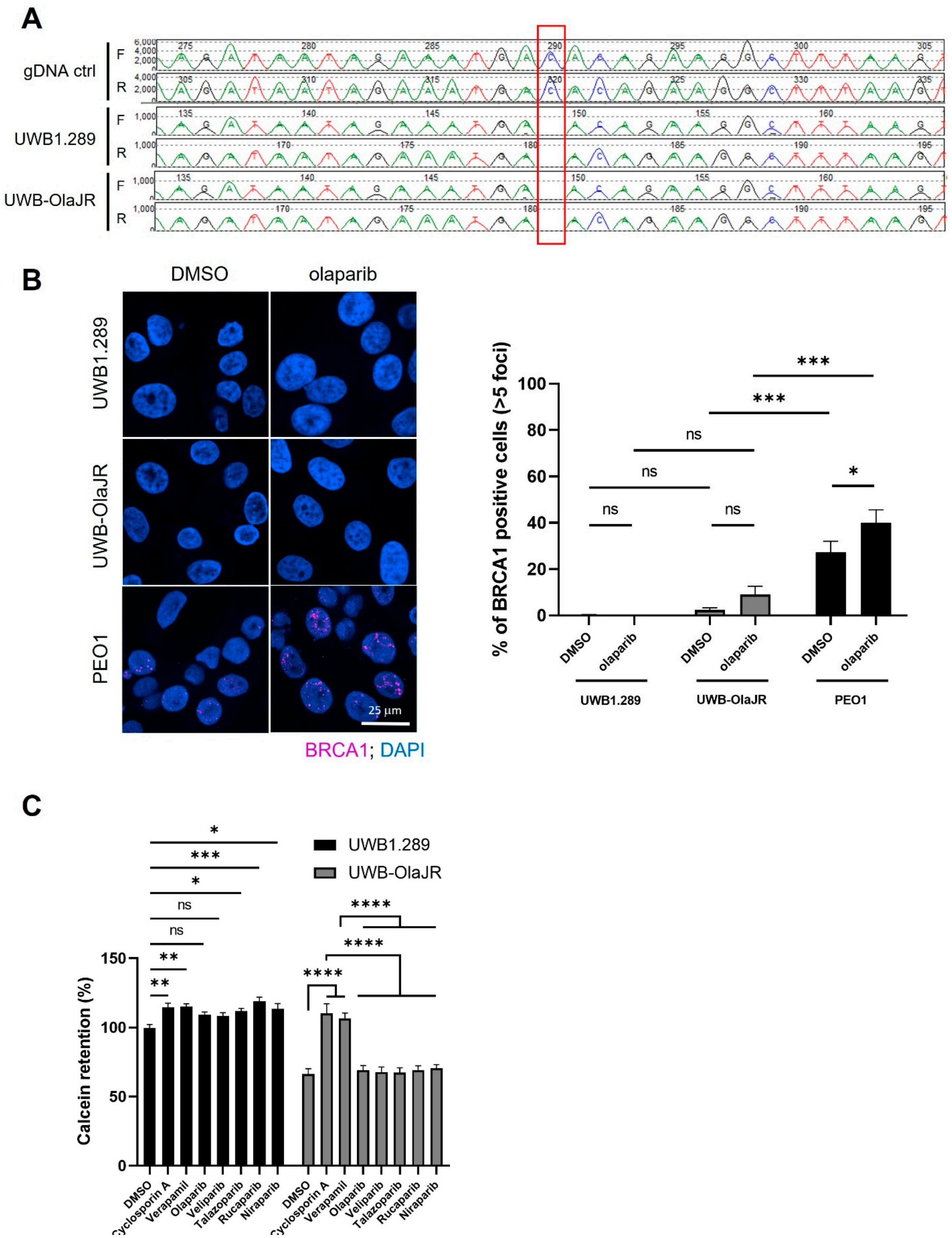

Supplementary Figure S1. UWB-OlaJR cells do not exhibit functional BRCA1 or increased multidrug resistance (MDR) activity when treated with PARPis. (A) Sanger sequencing of exon11 of BRCA1 in UWB1.289 and UWB-OlaJR. (B) Cells were treated with 10 μM of olaparib for 2 hours. Immunofluorescence staining of BCRA1 foci was performed to examine the expression. Representative images were taken at × 60 magnification. Cells with >5 BCRA1 foci were counted as BRCA1-positive (BRCA1 +) cells. The percentage of BRCA1+ cells is plotted. (C) MDR activity assay was used to compare MDR-dependent efflux of the substrate calcein in UWB1.289 and olaparib-resistant UWB-OlaJR in the presence of PARPis, cyclosporin A, and verapamil. ****, $p < 0.0001$; ***, $p < 0.001$; **, $p < 0.01$; *, $p < 0.05$; ns, not significant; one-way ANOVA.

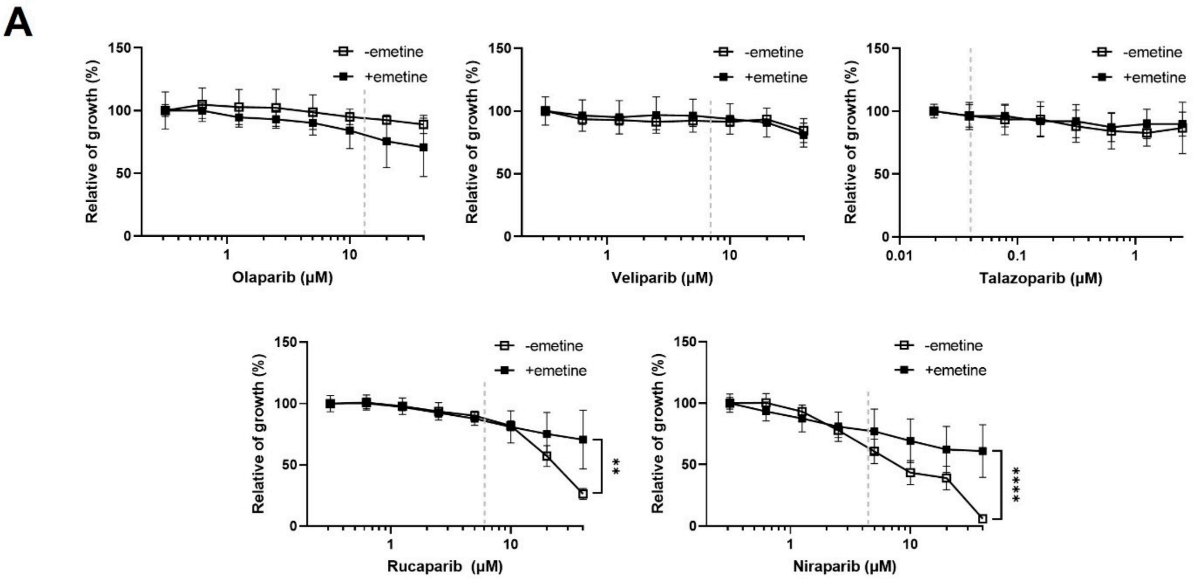

Supplementary Figure S2. Inhibition of DNA replication mitigates the cytotoxic effects of rucaparib and niraparib. (A) UWB-OlaJR cells were incubated with 10 μM emetine for 30 minutes and washed away then, followed by treatment of olaparib, veliparib, talazoparib, niraparib, and rucaparib at indicated doses for 5 days. The value 0 was plotted as 0.0195 μM in talazoparib and 0.3125 μM in veliparib, niraparib, and rucaparib for better visualization, since 0 cannot be plotted on logarithmic scale. All experiments were repeated in triplicate. Data are shown as mean ± SD and. IC50 values were calculated using GraphPad Prism v9. ****, $p < 0.0001$; **, $p < 0.01$; ns, not significant; student's t-test.

Supplementary Table S1. Damaging or deleterious genes identified in UWB-OlaJR by WES compared to UWB1.289.

Gene	Base change	Amino acid change	SIFT	PolyPhen
FRG2C	c.1280C>A	p.Pro427His	deleterious (0)	possibly damaging (0.701)
NPIPB12	c.1357G>C	p.Ala453Pro	deleterious (0)	probably damaging (0.959)
USP17L30	c.92A>G	p.Glu31Gly	deleterious (0)	probably damaging (0.999)
VPS28	c.1044G>T	p.Glu348Asp	deleterious (0)	probably damaging (0.986)
CLCNKB	c.2042T>G	p.Leu681Arg	deleterious (0)	probably damaging (0.999)
EFHB	c.392T>A	p.Ile131Asn	deleterious (0)	possibly damaging (0.574)
MIA3	c.425T>C	p.Val142Ala	deleterious (0.04)	probably damaging (0.999)
TMEM209	c.250A>T	p.Ser84Cys	deleterious (0)	possibly damaging (0.726)
GRAMD1B	c.1099C>G	p.Leu367Val	deleterious (0)	probably damaging (0.919)
ADCY7	c.695C>G	p.Ser232Cys	deleterious (0)	probably damaging (0.945)
AGO1	c.2690C>A	p.Pro897His	deleterious (0.02)	probably damaging (0.969)
AGO1	c.4297G>C	p.Val1433Leu	deleterious (0.01)	probably damaging (0.998)
ANKRD36C	c.1138T>G	p.Phe380Val	deleterious (0)	probably damaging (0.977)
ANO2	c.2738A>C	p.Lys913Thr	deleterious_low_confidence (0.02)	probably damaging (1)
ANO2	c.1076C>T	p.Ala359Val	deleterious (0)	probably damaging (0.996)
BAZ2B	c.203T>A	p.Ile68Asn	deleterious (0)	probably damaging (0.997)
CACNA2D1	c.303G>C	p.Gln101His	deleterious (0.02)	possibly damaging (0.886)
CD38	c.219G>A	p.Met73Ile	deleterious (0.03)	possibly damaging (0.5)
CDH24	c.185A>C	p.Gln62Pro	deleterious (0)	possibly damaging (0.804)
EHMT1	c.703C>T	p.Arg235Cys	deleterious (0)	probably damaging (0.998)
EIF4A2	c.233T>G	p.Ile78Ser	deleterious (0)	probably damaging (1)
EIF4A2	c.7701A>C	p.Glu2567Asp	deleterious (0.02)	possibly damaging (0.874)
EIF4A2	c.170G>A	p.Arg57His	deleterious (0)	probably damaging (1)
HCN2	c.1044G>T	p.Glu348Asp	deleterious (0)	probably damaging (0.986)
HMGA2	c.3685G>A	p.Glu1229Lys	deleterious (0)	probably damaging (0.994)
LRRC56	c.203T>A	p.Ile68Asn	deleterious (0)	probably damaging (0.997)
MRPS15	c.219G>A	p.Met73Ile	deleterious (0.03)	possibly damaging (0.5)
MS4A4A	c.185A>C	p.Gln62Pro	deleterious (0)	possibly damaging (0.804)
MYH15	c.2042T>G	p.Leu681Arg	deleterious (0)	probably damaging (0.999)

NCAM1	c.191T>G	p.Leu64Arg	deleterious (0.01)	possibly damaging (0.486)
RIMS3	c.498C>G	p.Ser166Arg	deleterious (0.02)	probably damaging (0.996)
RIMS3	c.479C>A	p.Ser160Tyr	deleterious (0.01)	probably damaging (0.949)
TMC2	c.223A>C	p.Ser75Arg	deleterious (0.02)	possibly damaging (0.522)
TRIM77	c.4786T>A	p.Phe1596Ile	deleterious (0.03)	possibly damaging (0.879)
ZSCAN30	c.3029A>C	p.Glu1010Ala	deleterious_low_confidence (0.05)	probably damaging (0.989)
CCDC57	c.425T>C	p.Val142Ala	deleterious (0.04)	probably damaging (0.999)
CFB	c.80C>T	p.Ala27Val	deleterious (0)	possibly damaging (0.795)
CTC1	c.250A>T	p.Ser84Cys	deleterious (0)	possibly damaging (0.726)
DNAH12	c.191T>G	p.Leu64Arg	deleterious (0.01)	possibly damaging (0.486)
DRC1	c.1357G>C	p.Ala453Pro	deleterious (0)	probably damaging (0.959)
FBXL19	c.5G>A	p.Gly2Glu	deleterious_low_confidence (0)	possibly damaging (0.728)
GPR20	c.92A>G	p.Glu31Gly	deleterious (0)	probably damaging (0.999)
GTPBP1	c.3143C>T	p.Thr1048Ile	deleterious (0)	probably damaging (0.998)
HLA-A	c.3029A>C	p.Glu1010Ala	deleterious_low_confidence (0.05)	probably damaging (0.989)
HLA-DQA1	c.33G>T	p.Glu11Asp	deleterious_low_confidence (0.02)	possibly damaging (0.899)
KIR3DL1	c.2690C>A	p.Pro897His	deleterious (0.02)	probably damaging (0.969)
KIR3DL1	c.4297G>C	p.Val1433Leu	deleterious (0.01)	probably damaging (0.998)
KIR3DL1	c.1203T>G	p.Ile401Met	deleterious (0)	possibly damaging (0.824)
KIR3DL2	c.113G>T	p.Gly38Val	deleterious (0)	probably damaging (0.984)
KRT3	c.1138T>G	p.Phe380Val	deleterious (0)	probably damaging (0.977)
KRT3	c.1076C>T	p.Ala359Val	deleterious (0)	probably damaging (0.996)
MED13L	c.303G>C	p.Gln101His	deleterious (0.02)	possibly damaging (0.886)
MUC6	c.80C>T	p.Ala27Val	deleterious (0)	possibly damaging (0.795)
NXF1	c.7701A>C	p.Glu2567Asp	deleterious (0.02)	possibly damaging (0.874)
OR8K1	c.5G>A	p.Gly2Glu	deleterious_low_confidence (0)	possibly damaging (0.728)
OR8K1	c.810T>G	p.Ser270Arg	deleterious_low_confidence (0.02)	probably damaging (0.933)
OTOF	c.1280C>A	p.Pro427His	deleterious (0)	possibly damaging (0.701)
PARS2	c.1688C>G	p.Ala563Gly	deleterious (0.01)	probably damaging (0.99)
PARS2	c.92A>G	p.Glu31Gly	deleterious (0)	probably damaging (0.999)
PCDH7	c.1099C>G	p.Leu367Val	deleterious (0)	probably damaging (0.919)
PDIA4	c.3143C>T	p.Thr1048Ile	deleterious (0)	probably damaging (0.998)
PLXNA2	c.3029A>C	p.Glu1010Ala	deleterious_low_confidence (0.05)	probably damaging (0.989)

PMS1	c.1669C>A	p.Pro557Thr	deleterious (0)	possibly damaging (0.829)
PRUNE2	c.3685G>A	p.Glu1229Lys	deleterious (0)	probably damaging (0.994)
SAMD9L	c.2690C>A	p.Pro897His	deleterious (0.02)	probably damaging (0.969)
SCUBE2	c.1203T>G	p.Ile401Met	deleterious (0)	possibly damaging (0.824)
SECISBP2L	c.113G>T	p.Gly38Val	deleterious (0)	probably damaging (0.984)
SSPO	c.1673T>G	p.Leu558Trp	deleterious (0)	probably damaging (0.999)
SSPO	c.185A>C	p.Gln62Pro	deleterious (0)	possibly damaging (0.804)
TRBV6-6	c.11885A>C	p.Lys3962Thr	deleterious (0.01)	possibly damaging (0.558)
TRBV6-6	c.233T>G	p.Ile78Ser	deleterious (0)	probably damaging (1)
TRBV6-6	c.7701A>C	p.Glu2567Asp	deleterious (0.02)	possibly damaging (0.874)
TSPYL6	c.1280C>A	p.Pro427His	deleterious (0)	possibly damaging (0.701)
USP17L30	c.114A>C	p.Lys38Asn	deleterious (0.01)	possibly damaging (0.614)
VAT1L	c.548T>C	p.Leu183Pro	deleterious (0.05)	probably damaging (0.953)
VAT1L	c.3143C>T	p.Thr1048Ile	deleterious (0)	probably damaging (0.998)
PHACTR1	c.1044G>T	p.Glu348Asp	deleterious (0)	probably damaging (0.986)
SPOPL	c.2794G>C	p.Arg932Thr	deleterious_low_confidence (0)	probably damaging (0.985)
SRGAP3	c.219G>A	p.Met73Ile	deleterious (0.03)	possibly damaging (0.5)
AL353747.1	c.1203T>G	p.Ile401Met	deleterious (0)	possibly damaging (0.824)
CNTNAP3B	c.223A>C	p.Ser75Arg	deleterious (0.02)	possibly damaging (0.522)
FGF9	c.810T>G	p.Ser270Arg	deleterious_low_confidence (0.02)	probably damaging (0.933)
HLA-A	c.1669C>A	p.Pro557Thr	deleterious (0)	possibly damaging (0.829)
ITGAX	c.479C>A	p.Ser160Tyr	deleterious (0.01)	probably damaging (0.949)
NSD2	c.703C>T	p.Arg235Cys	deleterious (0)	probably damaging (0.998)
NSD2	c.233T>G	p.Ile78Ser	deleterious (0)	probably damaging (1)
PNPLA7	c.33G>T	p.Glu11Asp	deleterious_low_confidence (0.02)	possibly damaging (0.899)
SCN4A	c.4297G>C	p.Val1433Leu	deleterious (0.01)	probably damaging (0.998)
TRBV6-6	c.1357G>C	p.Ala453Pro	deleterious (0)	probably damaging (0.959)
TRBV6-6	c.703C>T	p.Arg235Cys	deleterious (0)	probably damaging (0.998)
ANKRD36	c.113G>T	p.Gly38Val	deleterious (0)	probably damaging (0.984)
RNMT	c.1789T>A	p.Tyr597Asn	deleterious (0.02)	probably damaging (0.994)
